# Big data in financial risk management: evidence, advances, and open questions: a systematic review

**DOI:** 10.3389/frai.2025.1658375

**Published:** 2025-10-01

**Authors:** Leonidas Theodorakopoulos, Alexandra Theodoropoulou, Aristeidis Bakalis

**Affiliations:** Department of Management Science and Technology, Panepistemio Patron, Patras, Greece

**Keywords:** systemic risk, financial decision-making, data governance, digital transformation, Fintech

## Abstract

**Introduction:**

The intersection of big data analytics and financial risk management has spurred significant methodological innovation and organizational change. Despite growing research activity, the literature remains fragmented, with notable gaps in comparative effectiveness, cross-sectoral applicability, and the use of non-traditional data sources.

**Methods:**

Following the PRISMA 2020 protocol, a systematic review was conducted on 21 peer-reviewed studies published between 2016 and June 2025. The review evaluated the methodological diversity and effectiveness of machine learning and hybrid approaches in financial risk management.

**Results:**

The analysis mapped the relative strengths and limitations of neural networks, ensemble learning, fuzzy logic, and hybrid optimization across credit, fraud, systemic, and operational risk. Advanced machine learning techniques consistently demonstrated strong predictive accuracy, yet real-world deployment remained geographically concentrated, primarily in Chinese and European banking and fintech sectors. Applications involving alternative and unstructured data, such as IoT signals and behavioral analytics, were largely experimental and faced both technical and governance challenges.

**Discussion/conclusion:**

The findings underscore the scarcity of systematic benchmarking across risk types and organizational contexts, as well as the limited attention to explainability in current implementations. This review identifies an urgent need for comparative, cross-jurisdictional studies, stronger field validation, and open science practices to bridge the gap between technical advances and their operational impact in big data–enabled financial risk management.

## 1 Introduction

Financial markets and institutions today operate amid unprecedented volatility and complexity, driven by the proliferation of digital technologies and the relentless expansion of data (Theodorakopoulos et al., 2022). The inadequacy of legacy risk management frameworks has been laid bare by a string of high-profile disruptions, from cyberattacks and algorithmic trading shocks to the systemic reverberations of global crises. In this turbulent environment, the intersection of big data analytics and financial risk management has emerged as both a crucible of innovation and a source of unresolved tension, offering transformative potential but also surfacing profound methodological and practical challenges (Osman and El-Gendy, 2025).

### 1.1 Research gaps

Current research on big data applications in financial risk management exhibits persistent fragmentation, with most studies constrained by methodological insularity and narrow empirical scope. The literature overwhelmingly prioritizes technical innovation; benchmarks of novel machine learning models, ensemble methods, or optimization techniques, while seldom evaluating their comparative effectiveness across diverse financial risk categories or organizational contexts (Addy et al., 2024). Real-world validation is frequently sidelined, as empirical analyses rely on proprietary, simulated, or narrowly scoped datasets. The result is a conspicuous absence of robust evidence on scalability, deployment challenges, or cross-sectoral generalizability (Nguyen et al., 2023). Moreover, although the promise of integrating non-traditional data sources such as IoT (Internet of Things) streams, social media, or behavioral signals is often acknowledged, few studies operationalize this fusion in a manner that meaningfully advances predictive accuracy or risk governance. Equally striking is the literature’s neglect of explainability, interpretability, and practical adoption. Technical performance metrics are foregrounded, yet questions of model transparency, managerial usability, and regulatory compliance are largely unaddressed (Hilbert and Darmon, 2020). There is a near-total lack of cross-jurisdictional or comparative regulatory analysis, leaving the field ill-equipped to inform global best practices or anticipate sector-specific policy impacts.

This review directly addresses the most pressing and empirically tractable gaps by systematically mapping and synthesizing recent advances in: (i) the comparative strengths and limitations of major big data techniques across financial risk categories; (ii) the extent and nature of real-world deployment and scalability; (iii) the operationalization and impact of non-traditional data integration; and (iv) the influence of regulatory, geographical, and sectoral contexts on adoption and effectiveness. However, while the review highlights the critical importance of explainability, interpretability, and practical adoption, it recognizes that the current evidence base remains too limited to support a comprehensive synthesis or actionable guidance on these dimensions. By clarifying both the boundaries and the core contributions of this systematic review, the work aims to provide a transparent, critical foundation for both scholarly advancement and future research priorities in big data-enabled financial risk management.

### 1.2 Aim of the review and research questions

This review aims to systematically map, compare, and critically synthesize the recent empirical and conceptual advances in the application of big data analytics to financial risk management. The overarching goal is to clarify which big data-driven techniques are most effective across different risk types and sectors, to assess the extent of real-world deployment and scalability, to evaluate how the integration of non-traditional and unstructured data can enhance risk prediction, and to illuminate the influence of regulatory, geographical, and sectoral contexts. In pursuing these objectives, this review is guided by the following four research questions:

*RQ1: What are the comparative strengths, limitations, and practical trade-offs of different big data-driven analytical techniques (such as neural networks, ensemble machine learning, fuzzy logic, and information fusion) in managing various categories of financial risk (credit, fraud, systemic, and operational) across sectors?*

*RQ2: How do real-world applications of big data and AI models in financial risk management perform when deployed at scale, and what challenges or gaps remain regarding generalizability, data diversity, and integration with organizational processes?*

*RQ3: To what extent does the integration of non-traditional and unstructured data sources—such as IoT signals, social media, and behavioral analytics—enhance the predictive accuracy and early warning capabilities of financial risk models, and what barriers persist in achieving widespread adoption?*

*RQ4: What regulatory, geographical, or sectoral differences shape the adoption, effectiveness, and governance of big data techniques for financial risk management, and where do current studies fail to provide comparative or global perspectives?*

By addressing these questions through a rigorous synthesis of 21 recent studies, this review seeks to advance both scholarly understanding and practical innovation in big data-enabled financial risk management. Unlike prior reviews, which have tended to focus narrowly on either technical innovation or sector-specific case studies, this work offers a comprehensive, comparative mapping across methods, risk types, data modalities, and organizational contexts. By foregrounding not only algorithmic advances but also barriers to real-world adoption and cross-jurisdictional applicability, this review establishes a broader, more integrated agenda for both research and practice.

### 1.3 Structure of the paper

Charting a clear path through this multidisciplinary and rapidly evolving terrain requires both conceptual clarity and methodological precision. To that end, the paper is structured to guide the reader from foundational concepts to analytical synthesis and actionable insight. Section 2 establishes the conceptual framework, tracing the evolution of big data and its integration into financial risk management. Section 3 sets out the materials and methods, detailing the systematic review protocol, search strategy, and data extraction process. Section 4 presents the results, mapped to the four guiding research questions and reinforced by visual and tabular evidence. Section 5 engages in critical discussion, weaving together methodological, sectoral, and practical perspectives. Section 6 delineates the study’s limitations, while Section 7 articulates key priorities for future research. Finally, Section 8 concludes by distilling the review’s broader implications for scholarship, industry practice, and policy in the data-driven financial landscape.

## 2 Literature review

### 2.1 Big data: from 3 Vs to 8 Vs and beyond

Big data emerged not from theoretical abstraction, but from organizations’ desperate attempts to navigate a deluge of information that rendered traditional analytics and management tools obsolete. Early scholars and practitioners distilled this phenomenon into three foundational dimensions: Volume, capturing the unprecedented scale of digital records; Velocity, reflecting the relentless inflow of data that often arrives in real time or near real time; and Variety, signaling the proliferation of data types, from tidy relational tables to sprawling unstructured formats like text, images, and streaming sensor feeds. These original “3 *Vs*” offered a new vocabulary for a technological transformation, but practice soon revealed that the challenge was far deeper (Theodorakopoulos et al., 2024).

As digital ecosystems expanded and organizational ambition grew, so did the need to capture the full spectrum of big data’s complexity. The paradigm evolved, moving well beyond the initial triad to encompass five additional attributes: Veracity, acknowledging the constant struggle with data uncertainty, errors, and bias; Value, focusing analytical attention on extracting actionable insights and meaningful outcomes rather than collecting data for its own sake; Variability, recognizing the fluctuating meaning, structure, and context of data as it moves across systems and time; Visualization, underlining the importance of rendering complex analytical outputs comprehensible and usable for decision-makers; and Validity, the critical assurance that data and derived results are trustworthy, accurate, and truly reflect the phenomena under analysis. Together, these eight Vs encapsulate not only the technical hurdles of handling massive, messy, and dynamic datasets, but also the strategic, organizational, and epistemological imperatives now entwined with data-driven innovation (Ezzahra et al., 2019).

Today, the big data landscape is characterized by this multidimensionality. Organizations face not just a flood of data, but a tangled web of challenges around integrating unstructured sources, safeguarding accuracy and reliability, and bridging the gap between algorithmic power and managerial judgment. The rise of distributed computing frameworks, the proliferation of sophisticated machine learning architectures, and the automation of data preparation pipelines have redefined the art of the possible; yet the real advantage lies not in technical wizardry alone, but in the persistent work of transforming raw, multidimensional data into decisions that are both credible and consequential. In this evolving landscape, the 8 Vs serve less as a checklist than as a reminder that every new technical advance must still reckon with questions of meaning, context, and trust (Mumuni and Mumuni, 2024).

**Figure 1:**
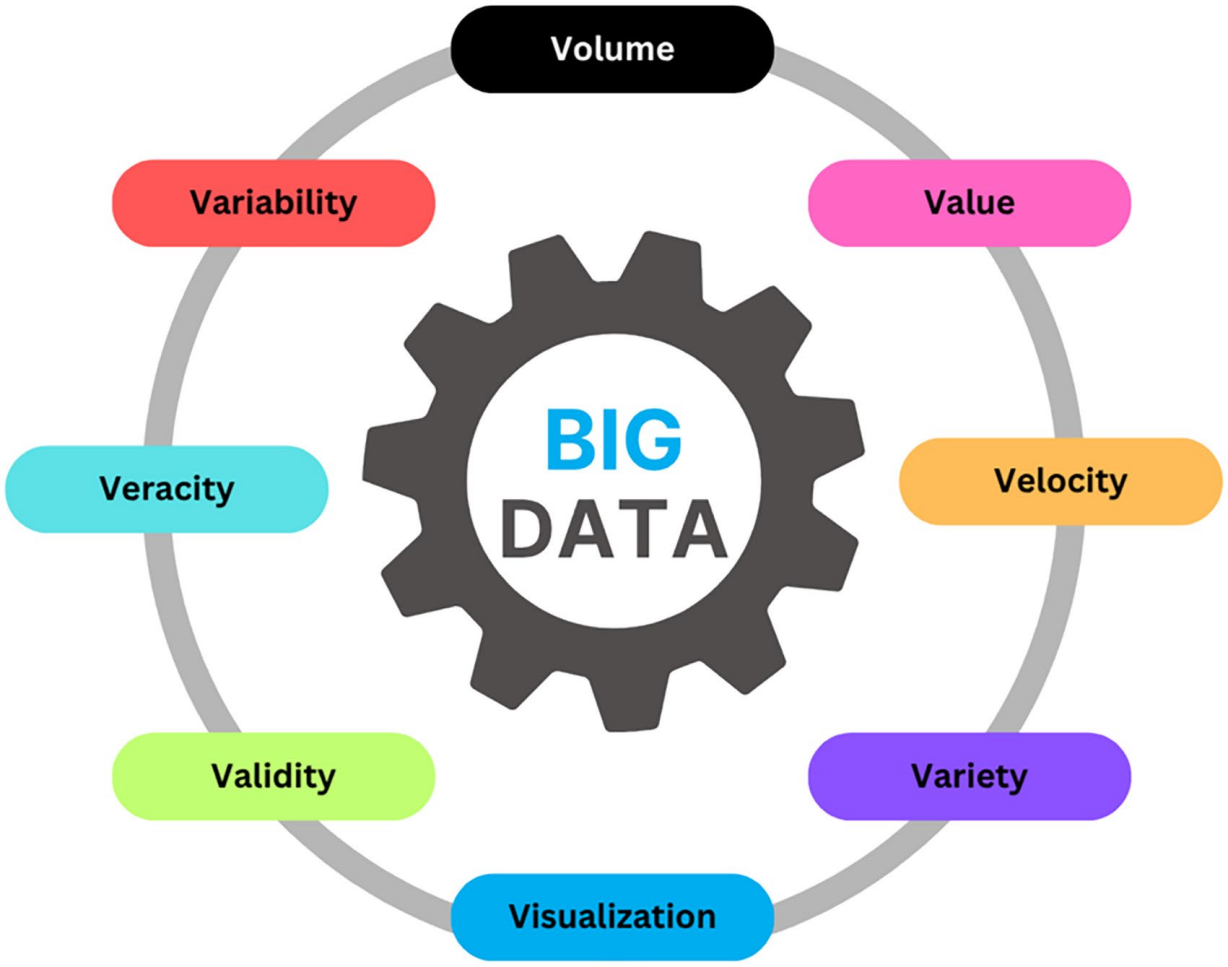
8 Vs of big data.

Finally, we make explicit that data vulnerability is intrinsic to big data in finance. Data are not only voluminous and heterogeneous, they are also targets and moving parts. They can be leaked, tampered with, or subtly poisoned at any stage of the pipeline, from collection to labeling to deployment. In this review we treat vulnerability as part of Veracity and Validity, and we use that lens when discussing provenance, access control, and continuous monitoring across later sections (Figure 1).

### 2.2 Financial risk management: core concepts and mitigation strategies

Financial risk management has long stood as a pillar of prudent organizational governance, a discipline defined by its relentless preoccupation with uncertainty and its commitment to safeguarding value in the face of volatility. At its core, the field is anchored in a cycle of anticipation, quantification, surveillance, and intervention; a perpetual process designed to identify risks before they crystallize into losses. Traditionally, risk managers have focused on a taxonomy that includes credit risk, the perennial challenge of counterparties defaulting on their obligations; market risk, the threat posed by unpredictable fluctuations in prices, rates, or currencies; operational risk, a sprawling category that encompasses breakdowns in processes, systems, and even human error; liquidity risk, which can leave otherwise healthy organizations paralyzed in times of stress; and systemic risk, the lurking specter of contagion that can transform local shocks into global crises (Huynh et al., 2025).

Mitigation of these diverse risks has given rise to a formidable toolkit. Statistical models, from classical regression to more intricate time series and copula-based approaches, have served as the analytical backbone for decades. Scenario analysis and stress testing have evolved into sophisticated exercises, allowing managers to peer into the fog of potential futures and assess resilience under extreme, if improbable, conditions. Value-at-risk (VaR), though not without controversy, remains a lingua franca for risk quantification, providing a common metric for both regulators and practitioners. Regulatory compliance frameworks, shaped by successive waves of Basel Accords and the increasingly intricate directives of the European Union and other bodies, now exert immense influence, not merely as constraints but as drivers of innovation in risk measurement and reporting. Insurance, derivatives, and risk transfer mechanisms form the second line of defense, while robust internal controls, governance structures, and audit practices round out the institutional response (Lentini and Thayasivam, 2025).

Yet, this traditional machinery is under growing strain. The past decade has forced risk managers to grapple with threats that defy familiar categorization: cyber risk, where attacks can materialize from distant actors and propagate invisibly across networks; reputational risk, magnified by the velocity and reach of digital media; and the emergent, tangled webs of interdependence that characterize modern financial ecosystems. The collapse of a single node, whether due to fraud, error, or malfeasance, can ripple outward, challenging the very assumptions upon which risk models were built. At the same time, the regulatory environment has become both more demanding and more dynamic. Basel III, for example, has not only tightened capital requirements and stress test protocols but also heightened expectations for data quality, model governance, and risk transparency. Parallel initiatives in data privacy and digital finance are raising the bar for how risk data is managed, reported, and secured (González García and Álvarez-Fernández, 2022).

Against this backdrop, the role of risk analytics is being fundamentally reimagined. Static, backward-looking models are being displaced by agile, real-time analytics that can ingest vast, heterogeneous datasets and generate early warning signals before conventional indicators even twitch. The competitive imperative is no longer just to comply, but to anticipate, adapt, and innovate, transforming risk management from a reactive function into a strategic engine for resilience and value creation. This transition, however, is far from straightforward; it brings its own risks, from model overfitting and opacity to data governance and ethical dilemmas. The next era of financial risk management will be shaped by how effectively organizations can navigate these new uncertainties, marrying technological capability with judgment, foresight, and a relentless commitment to trustworthiness (Rozony et al., 2024).

### 2.3 The intersection: big data in financial risk management

The meeting point of big data analytics and financial risk management has become one of the defining frontiers in both domains—a collision of scale, speed, and sophistication that is fundamentally reshaping how organizations anticipate, interpret, and respond to risk. No longer bound by the limits of periodic reporting or narrow, siloed data streams, risk managers today can at least in theory draw from oceans of information: high-frequency market data, transactional histories, IoT device telemetry, social sentiment, and real-time macroeconomic signals. This vast, heterogeneous influx is neither orderly nor straightforward, yet it holds the raw potential to illuminate risks that once lurked in statistical shadows (Kalashnikov and Kartbayev, 2024). At the same time, this capability comes with a wider attack and failure surface. When models ingest streaming or unstructured sources, vulnerability becomes a design constraint rather than a peripheral concern, affecting feature stability, provenance, and the trust placed in any downstream decision.

Machine learning, deep learning, and other AI-powered methods have dramatically altered the risk analytics landscape. Rather than relying solely on regression or scenario-based stress tests, contemporary approaches can identify subtle patterns, anomalies, or outlier behaviors that might signal emerging fraud, credit deterioration, or liquidity shortfalls often surfacing weak signals invisible to classic models. The embrace of neural networks and ensemble techniques has made it possible to process and learn from both structured records and unstructured data, unlocking predictive and diagnostic capabilities at a scale previously unthinkable. Network analytics, meanwhile, now enable the mapping of interdependencies and contagion pathways within and across institutions, revealing vulnerabilities that are systemic rather than merely idiosyncratic. Information fusion bringing together disparate data sources further amplifies analytic depth, supporting early-warning systems and dynamic risk scoring in environments of profound uncertainty (Udeh et al., 2024).

At the same time, big data analytics have injected a distinct behavioral and sentiment-driven layer into the architecture of risk management, gradually challenging the long-standing dominance of hard financial ratios and macroeconomic indicators. For the first time, market sentiment, the rhythm of public discourse, and subtle inflections in consumer behavior can be systematically quantified, tracked, and mapped to evolving risk profiles. This infusion of “soft data” promises explanatory insights that classic numerical models could not approach, allowing for earlier detection of market shifts or emerging crises fueled by rumor, panic, or collective exuberance. Yet this expansion is not without cost; questions of causality become knottier, as signals drawn from news feeds or social platforms can be clouded by noise, bias, and fleeting trends. The interpretability of such hybrid models, blending qualitative nuance with quantitative rigor, remains fiercely debated, as organizations confront the possibility that algorithmic complexity might obscure, rather than clarify, critical risk signals (Tiwari et al., 2025).

The pursuit of more holistic intelligence, blending structured fundamentals with a mosaic of alternative data sources, is now a focal point of both academic inquiry and industry innovation. It is precisely in this context that the need for systematic synthesis becomes urgent. The literature is fragmented; some studies chase technical novelty, others focus on deployment, but few map the full landscape of methodologies, application domains, and practical challenges. As the boundaries of financial risk management blur and the appetite for predictive, multidimensional analytics grows, a rigorous, evidence-driven assessment of the field is essential. This imperative shaped the present review’s methodological design, motivating the adoption of a transparent, protocol-driven approach that could cut through the complexity, evaluate the comparative strengths of emerging techniques, and expose persistent gaps. The following section details the Materials and Methods underpinning this synthesis, setting out the systematic process by which relevant literature was identified, screened, and mapped in service of a clearer, more integrated understanding of big data’s role in the evolving risk management landscape.

## 3 Materials and methods

**Figure 2:**
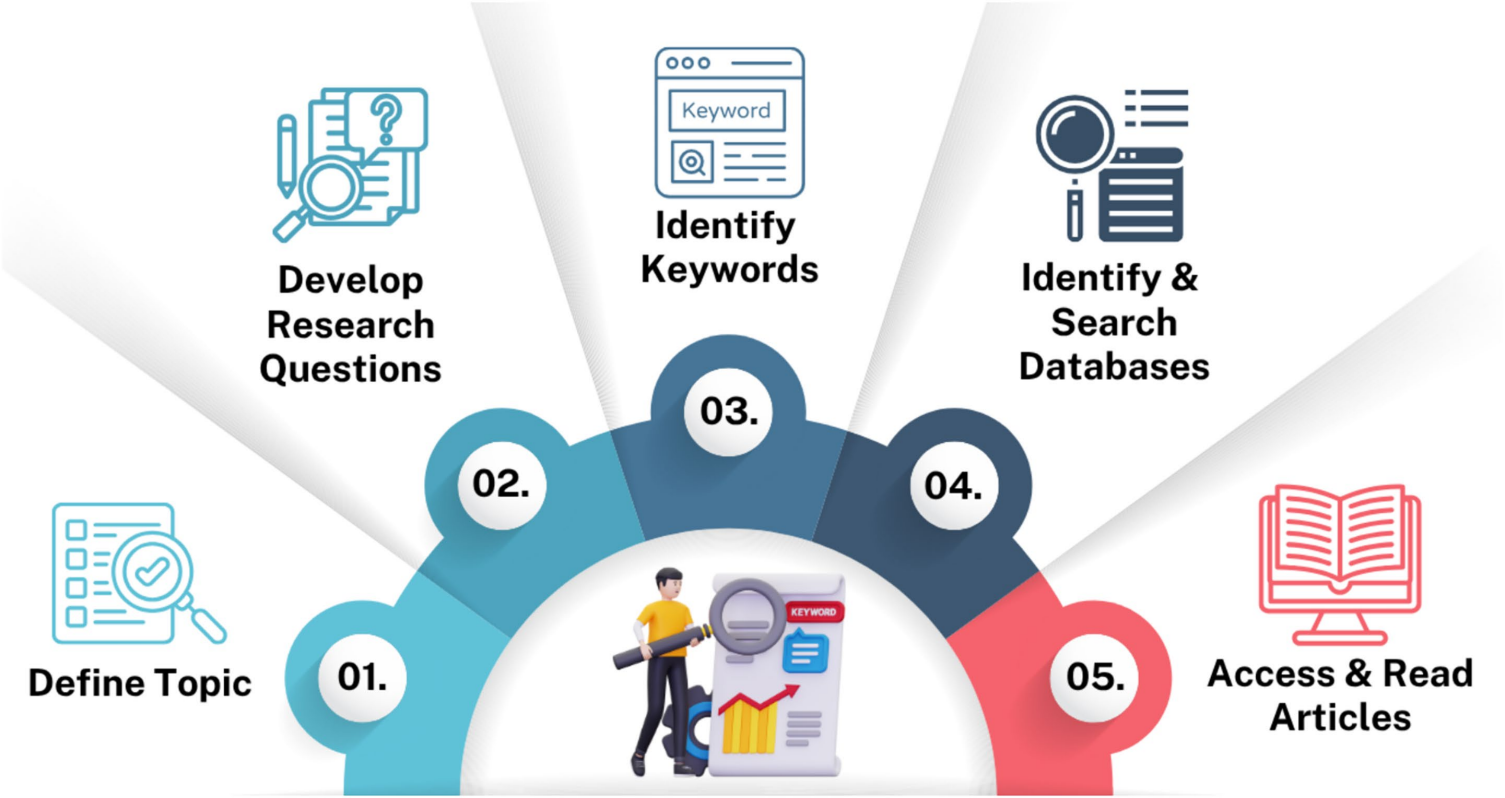
5-step PRISMA process.

This review adopts a systematic and transparent methodological framework, rooted in the PRISMA (Preferred Reporting Items for Systematic Reviews and Meta-Analyses) 2020 protocol (Page et al., 2021), to critically map the intersection of big data analytics and financial risk management. Recognizing the rapid methodological evolution and sectoral expansion of this research domain, our approach emphasizes both breadth and analytical depth. PRISMA 2020 is used to transparently document the review process and decisions as well as support the construction of a coherent evidence base capable of addressing both technical and contextual research questions. Methodological rigor was further reinforced by the use of a five-step process, visually summarized in Figure 2, that guided each phase from topic formulation to evidence extraction and synthesis. The review’s methodological design was explicitly tailored to surface not only dominant trends and high-performing algorithms, but also latent gaps, sectoral blind spots, and barriers to real-world impact.

All stages of the review process were executed sequentially and iteratively to maximize both coverage and relevance. The process began with the precise definition of the review’s thematic focus: the empirical and conceptual landscape of big data techniques in financial risk management across organizational and regulatory boundaries. Research questions were then developed, rooted in an initial scoping of the literature and aligned with recognized gaps in comparative methodology, deployment, data integration, and contextual adaptation. Comprehensive search strings were constructed, anchored by keywords such as “Big Data,” “Big Data Analysis,” “Machine Learning,” “Financial Risk Management” and “Systemic Risk,” and systematically applied across major scholarly databases. Anchoring on “Big Data” was deliberate: the review’s aim is to map work that self-identifies with this term in financial risk management, a corpus we found to be thin and fragmented relative to the broader Machine Learning (ML) literature. The Scopus database served as the primary repository, employing the following search syntax to ensure retrieval of peer-reviewed, English-language journal articles published between 2016 and June 2025:

*TITLE-ABS-KEY ((“Big Data” OR “Big Data Analysis”) AND (“Machine Learning”) AND (“Financial Risk Management” OR “Systemic Risk”)) AND PUBYEAR > 2015 AND PUBYEAR < 2026 AND (LIMIT-TO (LANGUAGE, “English”)) AND (LIMIT-TO (DOCTYPE, “ar”)).*

To further broaden coverage and capture relevant computer science research, equivalent queries were subsequently executed in the DBLP database. All identified articles underwent a multi-stage screening process, beginning with title and abstract review for initial relevance, followed by full-text reading and methodological mapping. This allowed for the rigorous identification of empirical, theoretical, and review studies that addressed at least one dimension of the guiding research questions. Only articles meeting strict quality and topicality criteria were retained for in-depth analysis and evidence synthesis.

### 3.1 Inclusion criteria

The following Inclusion Criteria were established to ensure the methodological rigor and thematic relevance of the studies selected for review (Table 1).

**Table 1:** Inclusion criteria of this SLR study.

#	Inclusion criteria
1	Published between January 2016 and June 2025
2	Written in the English language
3	Contains the keywords “Big Data,” “Big Data Analysis,” “Machine Learning,” “Financial Risk Management” and “Systemic Risk” in the title, abstract, or keywords section.

### 3.2 Exclusion criteria

The exclusion criteria were applied to remove studies that did not align with the review’s temporal, linguistic, or topical scope (Table 2).

**Table 2:** Exclusion criteria of this SLR study.

#	Exclusion criteria
1	Published before January 2016 or after June 2025
2	Not written in English
3	Does not contain the keywords “Big Data,” “Big Data Analysis,” “Machine Learning,” “Financial Risk Management” and “Systemic Risk” in the title, abstract, or Keywords section.

### 3.3 Study selection process

After removal of duplicate records, all retrieved articles were screened sequentially according to the inclusion and exclusion criteria. Initial screening was conducted on titles and abstracts to eliminate irrelevant or off-topic works. Remaining papers underwent full-text review to confirm eligibility and topical relevance. The overall flow of study selection, including the number of records identified, screened, included, and excluded at each stage, is detailed in Figure 2, which presents the PRISMA 2020 flow diagram for this review.

### 3.4 Data extraction and mapping

For each article retained after full-text screening, a structured data extraction protocol was implemented. Key variables extracted included authorship, publication year, sector and geographic context, type of data and big data technique (s) used, risk category addressed, methodological approach, key results, and reported limitations. This standardized mapping enabled both narrative and comparative synthesis across all studies, facilitating alignment with the review’s research questions and supporting evidence-based tabulation (see Table 3). Data extraction and coding were managed using Microsoft Excel. Where bibliometric or keyword co-occurrence analysis was relevant, VOSviewer was considered as the primary tool for network visualization and mapping. Visuals such as the PRISMA flow diagram were designed using Canva.

**Table 3:** Studies identified through the PRISMA 2020 protocol.

#	Authors/years	Title	Data/Context	Big data technique(s)	Risk type(s)	Analytical approach	Key results	Limitations
1	Shang et al. (2025)	A Robust Large-Scale Multi-Criteria Decision Algorithm for Financial Risk Management with Interval-Valued Picture Fuzzy Information	10 investment options, 7 criteria, expert-driven	Interval-valued picture fuzzy sets, MARCOS, MCGDM (Multi-Criteria Group Decision-Making)	Investment risk, market/credit/liquidity risk	Fuzzy Multi-Criteria Decision-Making (MCDM), aggregation operators	Robust under uncertainty, improves decision-making in volatile environments	Dependent on expert input, may lack big-data scalability
2	Xia et al. (2024)	A Novel Heuristic-Based Selective Ensemble Prediction Method for Digital Financial Fraud Risk	Three real-world digital financial fraud datasets	Selective ensemble (ENKMRH: K-means++, RILHHO), ensemble ML	Digital fraud risk (credit, lending, money laundering)	Heuristic ensemble, ML, optimization	Outperforms state-of-the-art, accuracy up to 93.8%, improves fraud risk prediction	May require extensive computational resources, focus on digital platforms
3	Deka et al. (2024)	Advanced Supply Chain Management Using Adaptive Serial Cascaded Autoencoder with LSTM and Multi-Layered Perceptron Framework	Financial risk in supply chain management, various industries	Adaptive autoencoder, LSTM, MLP, SGSO (Shapley-Guided Stochastic Optimization)	Supply chain financial risks	Deep learning hybrid, heuristic optimization	Outperforms GRU, MLP, AE-LSTM models (up to 10.9% better F1-score), robust for complex supply chains	Applied to supply chain finance, less focus on banking/market risks
4	Mani (2024)	An Exploration of Contemporary Trends in Finance Research	Content analysis of 160 finance publications (2018–2023)	Systematic content analysis, NVivo	Financial risk management, FinTech, digital finance, sustainable finance, etc.	Systematic review	Identifies key research areas and future trends, highlights tech-driven risks, calls for new analytics	Broad scope, not empirical, trend-oriented, less on methods
5	Cui and Yang (2024)	Optimization Algorithm for Enterprise Decision Making Based on Big Data Fusion	Enterprise decision-making, empirical application	Big data fusion, computer-aided optimization, BP neural network	Financial risk, enterprise risk	BP neural network, optimization algorithm	Optimized algorithm 20% more accurate than SVM; BP neural nets most stable for risk prediction	Focused on model performance, lacks field validation
6	Tayfor et al. (2024)	Optimized Deep Fuzzy Neural Network for Financial Risk Evaluation in Fintech Model	Fintech companies, German/Polish bankruptcy datasets	Deep fuzzy neural network, snake optimization, DBO	Financial risk, bankruptcy, fintech risk	Deep learning, multi-criteria decision-making	96–99% accuracy for risk classification in benchmark datasets	Simulation-based, not real sector/firm data
7	Singh et al. (2024)	What we know and what should we know about the future of blockchain in finance	500-article bibliometric analysis (global, 2018–2024)	VOSviewer, Bibliometrix, big data analytics, ML	Financial risk, blockchain, digital transformation	Bibliometric mapping, clustering	Highlights research frontiers—blockchain, big data, ML in risk management; identifies future research gaps	No empirical risk model, bibliometric review
8	Nivetha et al. (2024)	Exploring the Use of Big Data in Financial Risk Management and Fraud Detection	Kaggle credit card fraud dataset (Europe, 284,807 txns)	Random Forest, ML, Hadoop, Spark, XGBoost	Fraud risk, credit risk	Ensemble ML (RF, DT, XGBoost), big data analytics	RF model OOB score 0.933, AUC 0.978 for fraud detection in large-scale transactions	Focused on credit card fraud, limited to one dataset
9	Zhou (2023)	Financial risk assessment management of state-owned enterprises based on cloud accounting in the era of big data	State-owned enterprises, cloud accounting	Cloud accounting, fuzzy hierarchical analysis, evidence theory, neural networks, SVM (Support Vector Machine)	Financing, confidentiality, product/service risk	Fuzzy evaluation, risk scoring, multi-index system	Improves risk control via real-time cloud integration; dynamic risk assessment	Focus on SOEs, cloud/accounting context, not sector-wide
10	Murugan (2023)	Large-scale data-driven financial risk management & analysis using machine learning strategies	Large financial datasets, banks, IoT context	KNN, logistic regression, XGBoost, cluster analysis, IoT deployment	Credit risk, systemic risk, loan default	ML classification, cluster-based modeling, value-at-risk	XGBoost/KNN outperform baseline for credit risk; IoT boosts real-time insight	Model depends on large-scale data, requires advanced IT/IoT
11	Liu (2022)	Financial Risk Intelligent Early Warning System of a Municipal Company Based on Genetic Tabu Algorithm and Big Data Analysis	Listed companies; municipal China	Genetic tabu algorithm, big data analysis, CBR, SVM, neural networks	Early warning, credit/default, operational	Hybrid AI/ML early warning system	High accuracy, shortens warning time, real-time early warning, reduces crisis likelihood	Implementation complexity, focuses on listed/municipal companies
12	Wang and Wang (2022)	Internet Financial Risk Management in the Context of Big Data and Artificial Intelligence	Survey & empirical data from Chinese internet finance sector	Data analysis, AI algorithms, questionnaire analysis	Credit, operational, platform, regulatory risk	Empirical survey, descriptive analytics	Identifies key risks in internet finance; info security and law are top risk mitigators	China context, self-reported survey
13	Bi and Liang (2022)	Risk Assessment of Operator’s Big Data Internet of Things Credit Financial Management Based on Machine Learning	Case analysis of Company A, e-commerce, IoT environment	ML (logistic regression, decision tree), big data IoT	Credit, debt, operational, capital risk	Case study, ML classification	Machine learning improves credit risk assessment accuracy for IoT-based finance	Single company focus, limited generalizability
14	Yue et al. (2021)	Enterprise Financial Risk Management Using Information Fusion Technology and Big Data Mining	Enterprise sector, financial data from multiple firms	SVM, logistic regression, information fusion, big data mining	Enterprise financial risk	ML classification (SVM, LR), information fusion	Info fusion model achieves 95.18% accuracy, outperforming SVM and LR for risk classification	Limited to simulated/collected enterprise data
15	Cong (2021)	Research on Financial Risk Management of E-commerce Enterprises in the Era of Big Data	E-commerce, multiple platforms (China)	Big data analytics, AI, platform architecture	E-commerce financial risk	Strategic/theoretical analysis, platform modeling	Presents risk management strategies, builds architecture for e-commerce big data risk	Conceptual, lacks quantitative validation
16	Zhou et al. (2019)	A Big Data Mining Approach of PSO-Based BP Neural Network for Financial Risk Management With IoT	Chinese commercial bank, IoT-based chattel mortgage loans; on- & off-balance sheet data	PSO-based BP neural network, Apache Spark, Hadoop HDFS	Credit risk (default)	Parallel nonlinear optimization, AI/ML	Superior convergence, predictive accuracy, efficient screening of defaults, reduced processing time	Focused on one national context, only bank loans, limited to IoT data
17	Chakraborty (2019)	Evolving profiles of financial risk management in the era of digitization	Theoretical, industry-wide, digitization in finance	Big data analytics, machine learning, credit scoring automation	Credit, cyber, outsourcing, financial exclusion, macrofinance risk	Practitioner review, trend analysis	Explains digitization’s impact, new risk classes, regulatory challenges, and inclusion risks	Conceptual, not empirical, less on quantifiable models
18	Li (2018)	Design a management information system for financial risk control	Chinese banking/securities/trust sector; multi-institution context	SOM (Self-Organized Map) neural network, Hadoop, RFID, big data analytics	Systemic risk, credit risk, cross-infection risk	Real-time risk monitoring system, matrix-based assessment, automated controls	Real-time, automated, holistic risk monitoring; risk classification and dynamic response	China-specific context; depends on data integration and regulatory adoption
19	Srinivasan and Kamalakannan (2017)	Multi-Criteria Decision Making in Financial Risk Management with a Multi-objective Genetic Algorithm	Financial institutions, UCI ML benchmark datasets	MOGA (Multi-Objective Genetic Algorithm), business intelligence/data mining	Credit risk, enterprise risk, multi-criteria risk	Multi-objective genetic algorithm	MOGA enables improved multi-criteria decision-making in credit risk, better than classical methods	Tested on benchmark data, not sector-specific
20	Cerchiello and Giudici (2016a, 2016b)	Big data analysis for financial risk management	Systemic risk, interbank network, financial market + Twitter data	Graphical Gaussian models, semantic analysis, Bayesian fusion of big data	Systemic risk (contagion), bank failure	Graphical network models, market & social data fusion	First systemic risk model using both market and financial tweet data; improves insight into contagion	Data selection and preprocessing challenges, possible spurious signals from social data
21	Cerchiello and Giudici (2016a, 2016b)	Categorical network models for systemic risk measurement	Italian banking sector, financial market & Twitter data	Categorical graphical models, Bayesian data fusion, semantic tweet analysis	Systemic risk, contagion	Discrete/continuous graphical models, network analysis	First model combining tweet and market data for systemic risk; better mapping of interbank contagion	Reliance on tweet sentiment quality; context: Italian market

### 3.5 Quality assessment

We applied the Mixed Methods Appraisal Tool (MMAT; 2018) as a structured checklist adapted to algorithmic, non-interventional studies. Items not applicable were marked N/A; insufficient reporting was recorded as “Cannot tell.” We report item-level patterns and did not compute overall scores.

### 3.6 Synthesis approach

Data from eligible studies were synthesized using a hybrid narrative-comparative approach. Each paper was mapped to one or more of the guiding research questions, enabling cross-study comparison by technique, risk category, data type, and context. Major patterns, methodological trade-offs, and sectoral or geographical distinctions were identified both narratively and in summary tables. This multi-dimensional synthesis supported an integrated analysis of dominant trends, persistent gaps, and priorities for future research.

Before synthesis, we evaluated whether a quantitative meta-analysis was feasible. Given substantial heterogeneity in tasks, metrics, datasets, and incomplete variance reporting across studies, a formal meta-analysis was not appropriate. We therefore apply a structured narrative synthesis that contrasts techniques, risk domains, data regimes, and evaluation choices. Where three or more non-overlapping studies report the same task and metric, we summarize ranges descriptively without pooling. We also flag when included studies supply governance artefacts (e.g., documentation, oversight design, monitoring plans) aligned with emerging regulatory guidance, as these shape real-world deployability.

## 4 Results

The systematic review process resulted in a curated evidence base of 21 primary research articles, each critically examined to reveal methodological advances, sectoral trends, and enduring limitations in the deployment of big data analytics for financial risk management. The results are presented as an integrated narrative, closely tied to the PRISMA 2020 framework, and are substantiated throughout by a series of targeted visualizations designed to clarify both the process and the evolving landscape of this multidisciplinary domain.

**Figure 3:**
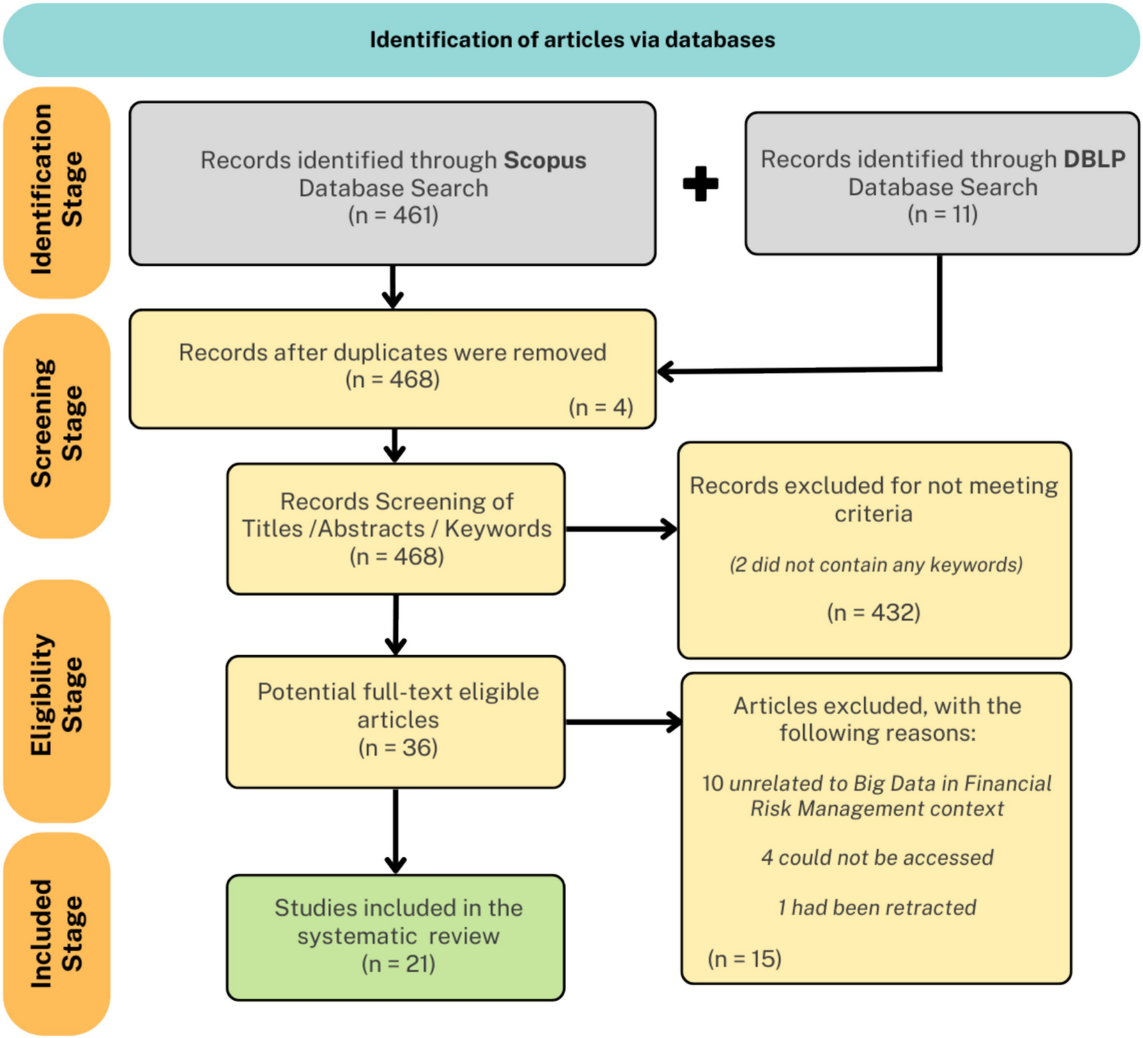
PRISMA flow diagram.

The foundation of this synthesis is established by the PRISMA 2020 flow diagram (Figure 3), which documents the multi-stage selection process applied to the initial pool of retrieved records. From a broad sweep of database search results, systematic application of inclusion and exclusion criteria, coupled with rigorous screening of titles, abstracts, and full texts, led to the exclusion of duplicates, non-English articles, and off-topic studies. Ultimately, this process distilled a large and heterogeneous initial corpus to a highly relevant set of empirical and conceptual studies, providing transparency and reproducibility in the construction of the final evidence base.

**Figure 4:**
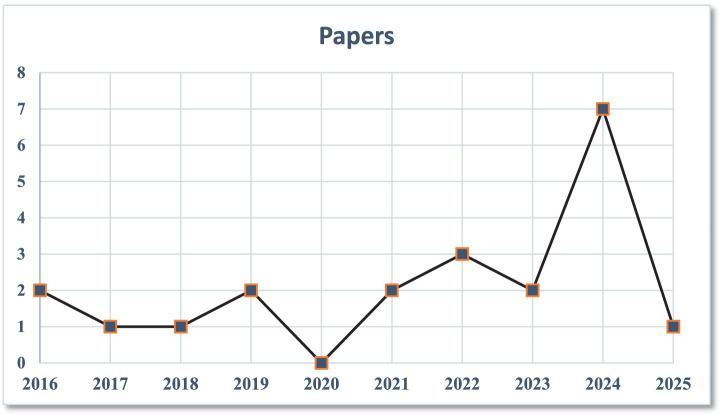
Number of studies published per year.

The temporal evolution of research activity within the field is depicted in Figure 4, which presents the distribution of included studies by year of publication. This visual underscores a dramatic escalation in scholarly output from 2021 onward, coinciding with the widespread adoption of machine learning and AI techniques in finance, the proliferation of digitized transaction data, and mounting global concern over systemic and cyber risks. The spike in recent years also reflects intensified academic and industry interest following high-profile incidents of financial disruption and regulatory transformation.

**Figure 5:**
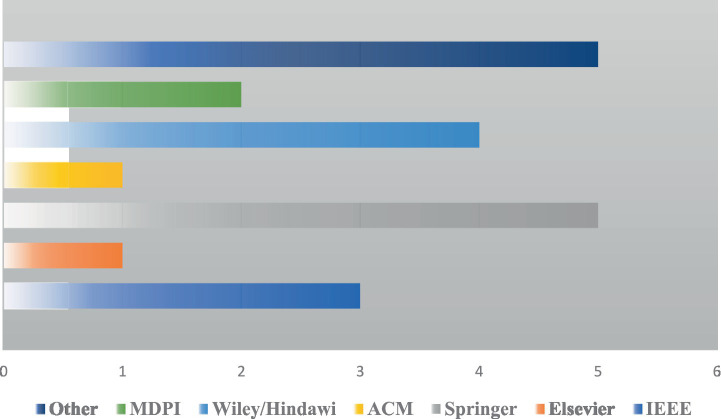
Number of studies published per publisher.

Figure 5 maps the distribution of studies by publisher, highlighting both the diffusion of research across major international publishing houses and the growing role of open-access platforms in disseminating methodological innovation. Notably, a significant proportion of the included literature appears in interdisciplinary journals and venues at the intersection of computer science, engineering, and finance, signaling the cross-domain migration of big data methods and the field’s accelerating convergence.

**Figure 6:**
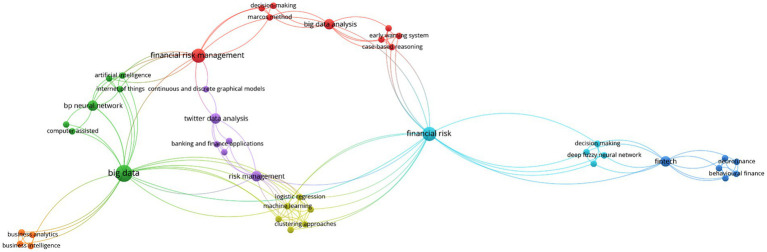
Co-occurrence keyword network.

A deeper layer of thematic structure emerges in Figure 6, which visualizes the keyword co-occurrence network derived from the included studies. This mapping reveals a dense and interconnected constellation of concepts, with prominent clusters centered on “machine learning,” “deep learning,” “fraud detection,” “credit risk,” “IoT,” and “blockchain.” The architecture of the network confirms that while a few analytic paradigms, particularly neural networks and ensemble learning, anchor the majority of empirical work, there is an increasing proliferation of hybrid methods and cross-cutting applications spanning domains from e-commerce to supply chain management. The visibility of terms such as “regulatory compliance,” “explainability,” and “unstructured data” at the network periphery also points to a latent research agenda around gaps and challenges yet to be systematically addressed.

These visuals provide a panoramic snapshot of the current evidence base, documenting not only the volume and distribution of research activity, but also the evolving methodological priorities and sectoral reach of the field. With this context established, the results are presented thematically according to the four guiding research questions, each supported by integrated narrative, summary tables, and direct cross-referencing to the mapped literature. This approach facilitates a transparent and comprehensive synthesis, aligning empirical patterns and research frontiers with the review’s overarching objectives.

Because outcome definitions and metrics are not commensurate across the corpus, we do not pool effects; instead we report structured comparisons and, when directly comparable, descriptive ranges.

To ground the subsequent thematic synthesis in methodological transparency and allow for precise cross-referencing, Table 3 presents a detailed mapping of the 21 studies included in this review. Each entry documents essential elements, such as authorship, publication year, analytical methodology, risk domain, sectoral or organizational context, and principal findings, enabling both a high-level overview and granular comparison across the evidence base. This tabular synthesis serves as both an analytical scaffold for the narrative discussion and a navigational aid for readers seeking to trace the evolution of big data approaches within specific financial risk categories, institutional settings, or methodological families. By foregrounding this structured evidence map, the review underscores its commitment to systematic rigor and supports transparent engagement with the breadth and diversity of recent scholarship at the intersection of big data and financial risk management.

To situate the evidence map in terms of study quality and reporting, we summarize an item-level appraisal using the Mixed Methods Appraisal Tool (MMAT; Version 2018 user guide) (Hong et al., 2018), adapted to algorithmic, non-interventional studies. Of the 21 items, 18 are empirical (quantitative non-randomized n = 15; quantitative descriptive n = 3) and 3 are conceptual/system pieces recorded as S2 = No and not domain-scored. Patterns are straightforward: measurement definitions and metrics are generally adequate (3.2 = Yes), while sampling frames, outcome completeness, and confounder handling are often under-reported (3.1/3.3/3.4 = mostly CT). Intervention adherence is inapplicable in this corpus (3.5 = N/A). We report distributions rather than any composite score, and use these ticks only to weight interpretation and claims of generalizability. Table 4 presents the MMAT summary (N/A = not applicable; CT = “cannot tell”).

**Table 4:** MMAT appraisal summary for the included studies.

Category of study designs	Methodological quality criteria	Responses			
		Yes	N/A	Cannot Tell	Comments
Screening Questions	S1. Are there clear research questions?	**X**			Applies to all 21 papers
	S2. Do the collected data allow to address the research questions?	**X**			Yes for 18 empirical; No for 3 conceptual/system — not domain-scored.
1. Qualitative	Is the qualitative approach appropriate to answer the research question?				No qualitative studies
	Are the qualitative data collection methods adequate to address the research question?				
	Are the findings adequately derived from the data?				
	Is the interpretation of results sufficiently substantiated by data?				
	Is there coherence between qualitative data sources, collection, analysis and interpretation?				
2. Quantitative randomized controlled trials	Is randomization appropriately performed?				No randomized studies
	Are the groups comparable at baseline?				
	Are there complete outcome data?				
	Are outcome assessors blinded to the intervention provided?				
	Did the participants adhere to the assigned intervention?				
3. Quantitative non-randomized	Are the participants representative of the target population?			**X**	Sampling frame/external validation rarely described
	Are measurements appropriate regarding both the outcome and intervention (or exposure)?	**X**			
	Are there complete outcome data?			**X**	Missingness/attrition seldom documented; often single static datasets.
	Are the confounders accounted for in the design and analysis?			**X**	Studies pursue prediction, not causal claims, so confounders typically not addressed.
	During the study period, is the intervention administered (or exposure occurred) as intended?				No assigned intervention in these designs.
4. Quantitative descriptive	Is the sampling strategy relevant to address the research question?			**X**	Bibliometric sampling frames implicit; survey frame not fully specified.
	Is the sample representative of the target population?			**X**	Representativeness not demonstrated.
	Are the measurements appropriate?	**X**			Variable/instrument definitions provided.
	Is the risk of nonresponse bias low?			**X**	N/A for bibliometric; Cannot tell for the survey (nonresponse not fully assessed).
	Is the statistical analysis appropriate to answer the research question?	**X**			Descriptive/inferential choices align with aims.
5. Mixed methods	Is there an adequate rationale for using a mixed methods design to address the research question?				N/A — no mixed-methods studies
	Are the different components of the study effectively integrated to answer the research question?				
	Are the outputs of the integration of qualitative and quantitative components adequately interpreted?				
	Are divergences and inconsistencies between quantitative and qualitative results adequately addressed?				
	Do the different components of the study adhere to the quality criteria of each tradition of the methods involved?				

*Given heterogeneous outcomes and incomplete variance reporting, no effect-size pooling was attempted.

Taken together, this appraisal gives us guardrails for interpretation rather than a scoreboard. Where studies report transparent data pipelines, suitable metrics, and some form of external validation, we treat their findings as more persuasive; where sampling frames, outcome completeness, or confounder handling are unclear, we read results as promising but provisional. These signals shape how we compare techniques and deployment contexts in the next section and help us flag what may or may not travel across jurisdictions, data regimes, and risk types. The Discussion uses these cues to weigh evidence, draw practical takeaways for financial risk management, and point to reporting practices that would materially strengthen the field (Table 4).

## 5 Discussion

The growing body of research on big data analytics in financial risk management is marked by methodological variety, contextual specificity, and uneven practical maturity. Given the heterogeneity and limited variance reporting in the primary studies, treating results as a quantitative meta-estimate would be misleading; our comparisons are therefore narrative and, where possible, anchored by descriptive ranges rather than pooled effects. While significant advances have been made in algorithm development, data integration, and early empirical applications, the field remains defined by persistent fragmentation and unresolved questions regarding comparative effectiveness, real-world impact, and transferability across domains. To provide a rigorous, evidence-based response to these challenges, the following sections synthesize findings from twenty-one recent studies, mapping both the state-of-the-art and the enduring limitations in the literature. The analysis is structured around four guiding research questions, each targeting a critical dimension of the current landscape: the comparative performance of big data techniques, their real-world deployment and scalability, the integration of non-traditional data sources, and the influence of regulatory and sectoral context. By addressing these questions systematically, the review aims to distill both actionable insights and priority areas for future scholarly and practical innovation.

Several use cases in our corpus (credit scoring/underwriting, fraud controls, some insurance risk models) fall within the EU AI Act’s high-risk scope, which triggers specific obligations on risk management, data governance, technical documentation/logging, transparency and human oversight, accuracy/robustness, conformity assessment and post-market monitoring. The Act entered into force on 1 Aug 2024 with phased application (selected prohibitions from Feb 2025; GPAI/governance rules from Aug 2025; most high-risk obligations fully applicable by Aug 2026–2027). For financial actors, this means treating model governance as a compliance-critical capability, not a research afterthought. In parallel, the OECD (Organization for Economic Co-operation and Development) AI Principles (human-centred values, transparency/explainability, robustness/safety, accountability) provide a pragmatic lens we can operationalize in FRM (Financial Risk Management) by (i) documenting provenance and feature lineage, (ii) reporting model cards and decision-support affordances for reviewers, and (iii) tracking fairness/impact metrics alongside utility. We reflect these requirements by reading empirical results as promising where governance artefacts exist, and as provisional where sampling, completeness or oversight are under-reported.

As big data pipelines increasingly ingest unstructured, behavioral, and real-time signals, vulnerability operates as a first-class property of the data itself, not just an external threat to be patched later. Data lineage breaks, adversarial contamination, and silent drift can alter distributions and semantics long before models see the inputs, which means that veracity, validity, and value are all conditional on how exposure to failure and attack is managed across the lifecycle. Treating vulnerability as intrinsic aligns the 8 Vs with contemporary practice, where provenance, resilience, and recoverability must be designed in from collection to consumption.

*RQ1: What are the comparative strengths, limitations, and practical trade-offs of different big data-driven analytical techniques (such as neural networks, ensemble machine learning, fuzzy logic, and information fusion) in managing* var*ious categories of financial risk (credit, fraud, systemic, and operational) across sectors?*

The landscape of big data-driven analytical techniques in financial risk management has evolved into a complex mosaic, reflecting the confluence of rapid technological progress and growing risk complexity across sectors. Recent years have witnessed an escalating shift from traditional statistical and rule-based models toward increasingly sophisticated paradigms, including advanced neural networks, ensemble learning, hybrid optimization, fuzzy multi-criteria systems, and network-based information fusion. Each of these paradigms responds to distinct practical pressures: the need for heightened predictive power in credit scoring and fraud detection, the imperative of robustness under uncertainty in market and investment risk, and the growing demand for systemic risk monitoring amid networked financial environments and contagion threats. Despite notable methodological progress, this literature remains shaped by sectoral silos, data accessibility, and the persistent tension between algorithmic accuracy, interpretability, and operational scalability. Benchmarking studies rarely cross boundaries between families of techniques or rigorously test their performance in diverse, real-world deployment scenarios. Instead, evidence is often context-bound; banking, fintech, or supply chain, relying on proprietary, simulated, or highly localized datasets. These conditions have produced a field where technical sophistication outpaces its integration into decision processes, and where the practical strengths and limitations of competing approaches remain underexplored at scale.

*Neural network-based approaches*, particularly those leveraging hybrid or optimized architectures, consistently demonstrate high predictive accuracy for credit risk, bankruptcy forecasting, and enterprise financial evaluation (Zhou et al., 2019; Liu, 2022; Tayfor et al., 2024; Cui and Yang, 2024; Bi and Liang, 2022; Yue et al., 2021). Particle Swarm-optimized Back Propagation (PSO-BP) neural networks, for instance, outperform conventional ML in processing IoT-driven loan data, with notable gains in both convergence and screening efficiency (Zhou et al., 2019). Deep fuzzy neural networks, particularly when enhanced by advanced optimization techniques, routinely achieve classification accuracies exceeding 95% on benchmark bankruptcy datasets, but such results are largely confined to simulation environments or proprietary data (Tayfor et al., 2024; Cui and Yang, 2024).

*Ensemble machine learning models*, notably Random Forest, XGBoost, and selective ensemble frameworks, dominate fraud detection, credit scoring, and operational risk applications, consistently outperforming classical single-model approaches (Xia et al., 2024; Nivetha et al., 2024; Murugan, 2023; Bi and Liang, 2022). Selective ensemble models (Xia et al., 2024) and XGBoost (Murugan, 2023) are shown to deliver superior robustness in highly imbalanced digital financial datasets, offering both high recall and computational scalability. However, these approaches often demand significant data preprocessing, feature engineering, and substantial computational resources, limiting ease of adoption in resource-constrained environments (Xia et al., 2024; Murugan, 2023).

*Fuzzy logic and multi-criteria decision-making (MCDM) techniques* find their greatest traction in contexts of investment, market, and liquidity risk, where uncertainty and conflicting stakeholder priorities are paramount (Shang et al., 2025; Zhou, 2023; Srinivasan and Kamalakannan, 2017). Interval-valued picture fuzzy sets and advanced aggregation operators, when combined with large-scale expert input, enhance robustness in volatile environments (Shang et al., 2025), but their scalability to fully automated, data-driven regimes remains limited.

*Network-based and information fusion models*, such as categorical graphical models and Bayesian data fusion, offer unique capabilities for systemic risk identification, especially by integrating non-traditional data sources like social media and interbank exposures (Cerchiello and Giudici, 2016a, 2016b). These approaches unlock new dimensions of market sentiment analysis and early warning, but their performance is sensitive to the quality and reliability of unstructured input, and practical deployment is often restricted to specific regulatory or market settings.

Lastly, *Hybrid and optimization-driven frameworks,* including genetic tabu algorithms, swarm optimization, and multi-objective evolutionary models, excel at early warning and multi-criteria decision tasks, adapting flexibly to complex organizational contexts (Liu, 2022; Deka et al., 2024; Srinivasan and Kamalakannan, 2017). Yet, the challenge of model transparency and the interpretability of results remains a significant barrier for managerial adoption (Table 5).

**Table 5:** Summary of techniques used in financial risk management.

Approach type	Key references	Core applications	Typical strengths	Typical limitations
Neural Networks and Deep Learning	Zhou et al. (2019), Liu (2022), Tayfor et al. (2024), Cui and Yang (2024), Bi and Liang (2022), and Yue et al. (2021)	Credit risk, bankruptcy, enterprise risk	High predictive accuracy; handles complex, nonlinear data	“Black box” nature, interpretability challenges, may require large datasets and tuning
Ensemble Machine Learning	Xia et al. (2024), Nivetha et al. (2024), Murugan (2023), and Bi and Liang (2022)	Fraud detection, credit scoring, ops risk	Superior performance on imbalanced data; robustness	High computational demand, complex preprocessing
Fuzzy Logic & MCDM	Shang et al. (2025), Zhou (2023), and Srinivasan and Kamalakannan (2017)	Investment, market, liquidity risk	Robust to uncertainty; models expert judgement	Limited automation, scalability to big data environments
Network-based & Information Fusion	Cerchiello and Giudici (2016a), Cerchiello and Giudici (2016b), and Yue et al. (2021)	Systemic risk, contagion, sentiment	Captures interconnectedness, integrates unstructured data	Sensitive to input quality, complex model calibration
Hybrid & Optimization Approaches	Liu (2022), Deka et al. (2024), and Srinivasan and Kamalakannan (2017)	Early warning, multi-criteria, adaptability	Flexible, adaptive to context, combines strengths	Model transparency and interpretability; field adoption

Taken together, these studies illustrate that no single big data technique universally outperforms others across all risk categories or sectors. Instead, the comparative strengths and limitations of each approach are shaped by domain requirements, data structures, and operational constraints. Neural networks and ensemble ML models dominate in predictive accuracy for well-structured risk tasks, while fuzzy MCDM and hybrid models are more robust under conditions of uncertainty and multi-stakeholder decision-making. Network and information fusion approaches are indispensable for systemic and contagion risk, particularly when alternative data streams, such as social media or network exposures, must be integrated. Hybrid and optimization-driven models provide flexibility and adaptability, excelling in complex, dynamic environments where early warning and multi-criteria evaluation are essential. Across all families of methods, however, the persistent trade-off between performance, interpretability, and practical scalability remains unresolved, underscoring the need for continued benchmarking, greater emphasis on transparent decision support, and more robust evidence from real-world deployments (Zhou et al., 2019; Xia et al., 2024; Shang et al., 2025; Cerchiello and Giudici, 2016a, 2016b; Liu, 2022; Tayfor et al., 2024; Nivetha et al., 2024; Murugan, 2023).

*RQ2: How do real-world applications of big data and AI models in financial risk management perform when deployed at scale, and what challenges or gaps remain regarding generalizability, data diversity, and integration with organizational processes?*

Despite the surge of algorithmic development in financial risk management, the real-world deployment and organizational integration of big data and AI models remain uneven and highly context-dependent. The literature reveals a sharp divergence between technical promise and practical impact: while a subset of studies demonstrate the feasibility of large-scale implementation and impressive predictive performance in operational settings, many contributions remain tethered to simulation environments, single-organization case studies, or limited-scope datasets. This landscape is characterized by a persistent tension between the scalability of advanced models, the diversity and heterogeneity of financial data streams, and the realities of integrating such systems into existing business processes and institutional routines.

Empirical evidence of successful, scalable deployment is strongest in studies leveraging substantial real-world datasets or operating within digitally mature financial environments. For instance, Zhou et al. (2019) showcase a Spark-based PSO-BP neural network deployed for IoT-enabled loan risk screening in a Chinese commercial bank, demonstrating both computational efficiency and domain-specific predictive value. Similarly, Murugan (2023) report on the application of ensemble ML algorithms, including KNN, logistic regression, and XGBoost, within large-scale banking and IoT contexts, highlighting the capacity of these systems to outperform conventional models when given access to extensive, heterogeneous transaction records. Nivetha et al. (2024) extend this evidence to the domain of fraud detection, deploying Random Forest and XGBoost on an open-access Kaggle dataset comprising nearly 300,000 credit card transactions and achieving high predictive accuracy in a high-velocity, real-world data environment.

Yet, the leap from technical validation to routine field deployment remains substantial. Multiple studies underscore significant challenges related to data quality, model transferability, and the practical integration of big data analytics with legacy IT infrastructure and risk governance processes (Li, 2018; Bi and Liang, 2022; Yue et al., 2021; Xia et al., 2024). For example, while Li (2018) develops a real-time risk control platform for multi-institutional Chinese finance, the system’s effectiveness is deeply contingent on regulatory support and organizational buy-in, variables that are rarely controlled or measured in empirical work. The majority of AI/ML deployment case studies, such as Bi and Liang’s (2022) risk assessment in IoT-driven e-commerce, remain bounded by single-firm, proof-of-concept contexts, limiting the generalizability of their results. Meanwhile, Liu (2022) and Deka et al. (2024) describe hybrid or optimization-driven models for early warning and supply chain risk, yet their field-testing is restricted to municipal enterprises or select industrial partners.

A common thread in these studies is the necessity of robust IT and data infrastructure for successful scale-up. Where banking and fintech institutions possess established data pipelines, model deployment and iterative learning are feasible (Murugan, 2023; Zhou et al., 2019). However, in less digitally mature sectors or geographies, integration with organizational routines, regulatory compliance, and workforce upskilling remain formidable barriers. Wang and Wang (2022) and Yue et al. (2021) highlight the importance of managerial engagement and regulatory alignment, yet both ultimately acknowledge that current evidence is patchy and context-specific, with successful implementation stories often failing to translate beyond their original institutional setting (Table 6).

**Table 6:** Real-world application and deployment of big data and AI models in financial risk management.

Authors/Year	Sector/Context	Deployment setting	Main findings/outcomes
Zhou et al. (2019)	Banking (China, IoT)	Commercial bank, operational	High predictive accuracy; Spark enables scalability
Murugan (2023)	Banking, IoT	Multi-bank/IoT pilot	Ensemble ML outperforms baselines
Nivetha et al. (2024)	Finance (fraud)	Real-world, open dataset	RF/XGBoost high accuracy, large-scale
Li (2018)	Finance (China)	Real-time monitoring system	System-wide integration, dynamic response
Bi and Liang (2022)	E-commerce, IoT	Firm-level pilot	ML improved risk assessment in IoT
Yue et al. (2021)	Enterprise finance	Enterprise, modeling	Info fusion increases classification accuracy
Xia et al. (2024)	Fintech (fraud)	Digital platforms	Ensemble ML robust, outperforms baseline
Deka et al. (2024)	Supply chain finance	Pilot/testbed, industrial	Hybrid models outperform GRU, MLP
Liu (2022)	Municipal finance (China)	Listed/municipal firms	Shorter warning time, crisis prevention
Wang and Wang (2022)	Internet finance (China)	Internet finance sector	Managerial and legal risk mitigators

These findings, put together, reveal a field where large-scale, real-world application of big data and AI for financial risk management is not only possible, but demonstrably valuable, when the right technical and organizational preconditions are met. However, substantial challenges persist in achieving broad generalizability, particularly regarding the transfer of models across heterogeneous organizations, geographies, and risk types. The persistent reliance on isolated case studies and benchmark datasets underscores the need for more systematic research on integration, scalability, and the organizational dynamics that enable (or inhibit) the practical impact of big data analytics in finance (Zhou et al., 2019; Li, 2018; Murugan, 2023; Bi and Liang, 2022; Xia et al., 2024; Yue et al., 2021; Nivetha et al., 2024; Deka et al., 2024; Liu, 2022; Wang and Wang, 2022).

*RQ3: To what extent does the integration of non-traditional and unstructured data sources—such as IoT signals, social media, and behavioral analytics—enhance the predictive accuracy and early warning capabilities of financial risk models, and what barriers persist in achieving widespread adoption?*

The integration of non-traditional and unstructured data sources, such as IoT device signals, social media streams, and e-commerce behavioral data, has been widely heralded as the next frontier for financial risk modeling. Yet, a systematic examination of recent studies reveals that while enthusiasm is high, substantive operationalization remains the exception rather than the rule. Across the reviewed literature, only a handful of empirical works move beyond theoretical endorsement to actually implement or rigorously test the predictive value of heterogeneous data integration within financial risk management systems. Where realized, these approaches demonstrate clear, quantifiable improvements in both the timeliness and accuracy of risk prediction, particularly in domains where traditional financial indicators alone have proven insufficient.

Notably, Zhou et al. (2019) provide a compelling illustration of IoT data fusion in credit risk screening, leveraging a PSO-BP neural network architecture to analyze chattel mortgage loans in a Chinese commercial bank. Their model integrates sensor-derived asset and transaction data, resulting in significant gains in default prediction efficiency relative to conventional machine learning pipelines. A similar practical step forward is found in the work of Cerchiello and Giudici (2016a, 2016b), whose network-based models combine market financial information with semantic analysis of Twitter data to model systemic risk and contagion effects among Italian banks. By merging structured market metrics with real-time sentiment streams, their approach not only enhances early warning capabilities but also opens new avenues for capturing emerging, exogenous threats often overlooked by balance sheet analysis alone.

IoT-driven advances are also evident in Murugan (2023), where ensemble machine learning techniques, incorporating KNN, logistic regression, and XGBoost, are deployed on large, sensor-rich datasets in banking and enterprise contexts. Here, the incorporation of IoT telemetry is shown to boost real-time monitoring and model responsiveness, especially in environments characterized by rapid data velocity and volume. Bi and Liang (2022) reinforce these findings at the firm level, demonstrating that machine learning models which incorporate IoT operator data and e-commerce behavioral signals outperform those trained exclusively on standard financial attributes, particularly in predicting credit and operational risks (Table 7).

**Table 7:** Integration of non-traditional and unstructured data in financial risk management.

Authors/Year	Type of non-traditional data	Integration approach/model	Application domain	Main empirical outcome
Zhou et al. (2019)	IoT (sensor, transaction data)	PSO-BP neural network	Banking (credit risk)	Improved default prediction and screening efficiency
Cerchiello and Giudici (2016a)	Financial market + Twitter (sentiment analysis)	Graphical Gaussian models	Systemic risk (Italy, banking)	Enhanced early warning, contagion modeling
Cerchiello and Giudici (2016b)	Market data + Twitter (semantic)	Categorical graphical models	Systemic risk, contagion	Integration of social data improves systemic risk signals
Murugan (2023)	IoT telemetry, large-scale bank data	Ensemble ML (KNN, XGBoost, clusters)	Banking, enterprise	IoT data increases model responsiveness and accuracy
Bi and Liang (2022)	IoT operator, e-commerce behavior	ML (logistic regression, decision tree)	E-commerce, credit/ops risk	ML with IoT/behavioral data outperforms classic models
Singh et al. (2024)	Big data, IoT, blockchain (trend mapping)	Bibliometric mapping, clustering	Multiple sectors	Identifies rise of IoT/blockchain in literature
Cong (2021)	E-commerce, multi-source enterprise	Strategic/platform modeling	E-commerce, enterprise risk	Theorizes benefits and challenges of big data integration

While these studies highlight the transformative potential of unstructured and non-traditional data, the broader literature still largely defaults to classic financial variables or treats new data types as peripheral supplements. Bibliometric and conceptual syntheses, such as those by Singh et al. (2024) and Cong (2021), trace the emergence of big data, blockchain, IoT, and behavioral analytics as rising research trends but simultaneously note the field’s lag in operationalizing truly multimodal risk frameworks. In most cases, technical, organizational, and data governance barriers, including the lack of standardized data integration pipelines, data privacy concerns, the complexity of real-time unstructured data processing, and the absence of regulatory clarity, are cited as major obstacles to widespread adoption and scalable impact (Singh et al., 2024; Cong, 2021).

Thus, while select pioneering studies provide tangible evidence that the fusion of non-traditional and unstructured data sources can materially improve risk detection and early warning performance, the financial risk management literature as a whole remains in the early stages of this transition. Systematic, sector-wide adoption, and robust, cross-organizational validation, remains a frontier rather than a settled reality. The next wave of research and practice must focus on overcoming integration challenges, developing interpretable and regulatory-compliant architectures, and providing empirical benchmarks that move beyond isolated pilots (Zhou et al., 2019; Cerchiello and Giudici, 2016a, 2016b; Murugan, 2023; Bi and Liang, 2022; Singh et al., 2024; Cong, 2021).

*RQ4: What regulatory, geographical, or sectoral differences shape the adoption, effectiveness, and governance of big data techniques for financial risk management, and where do current studies fail to provide comparative or global perspectives?*

The diffusion and impact of big data techniques in financial risk management are inextricably linked to regulatory environments, national contexts, and sector-specific demands. Across the reviewed literature, a pronounced geographical skew emerges: the majority of empirical and applied research is anchored in the Chinese financial system or in highly localized case studies, with only sporadic forays into European (notably Italian) banking or global bibliometric mapping. As a result, the capacity to draw robust, cross-jurisdictional lessons or articulate sector-agnostic best practices remains fundamentally constrained.

Studies such as Zhou et al. (2019), Li (2018), and Wang and Wang (2022) ground their technical advances in the regulatory and institutional landscapes of China’s banking and internet finance sectors. Here, strong state-led digital infrastructure and proactive regulatory frameworks enable the rapid adoption of IoT-enabled risk models, real-time monitoring systems, and AI-driven fraud detection at scale. These deployments often benefit from centralized oversight, unified data standards, and comparatively streamlined approval processes, all of which are less prevalent in more fragmented or heterogeneous regulatory regimes.

By contrast, European contributions, most notably Cerchiello and Giudici (2016a, 2016b), focus on the integration of alternative data sources and network-based systemic risk models in the Italian banking context. While these studies are technically innovative, their practical deployment is shaped by the realities of EU data privacy regulations (such as GDPR), the complexity of cross-border banking supervision, and a generally more cautious, compliance-driven approach to big data adoption. This regulatory backdrop both constrains and refines the nature of data fusion and analytics used, foregrounding concerns around explainability, auditability, and consumer protection.

Sectoral divergence is also pronounced. In addition to the banking and fintech sectors, a smaller but growing body of work addresses e-commerce (Cong, 2021; Bi and Liang, 2022), supply chain finance (Deka et al., 2024), and state-owned enterprises (Zhou, 2023). These domains face unique data challenges and regulatory considerations; from the handling of transactional and behavioral data in digital commerce to the public accountability and audit requirements of government-linked entities. Notably, the adoption trajectory in these sectors is heavily influenced by sector-specific compliance standards, legacy IT integration hurdles, and differing levels of risk appetite and innovation readiness.

Bibliometric and conceptual analyses (Singh et al., 2024; Chakraborty, 2019) further illuminate the global landscape, mapping regional hotspots of big data innovation and regulatory engagement. These studies reveal that while the rhetoric of digital transformation and AI-driven risk governance is nearly universal, substantive differences persist in legal environments, data access, and institutional support; often reinforcing rather than bridging regional and sectoral divides (Table 8).

**Table 8:** Geographical, sectoral, and regulatory contexts in big data financial risk management studies.

Authors/Year	Region/Country	Sector/Domain	Key regulatory/contextual focus
Zhou et al. (2019)	China	Banking, IoT	State-led digital infrastructure, regulatory support
Li (2018)	China	Multi-sector finance	Real-time monitoring, regulatory integration
Wang and Wang (2022)	China	Internet finance	Legal compliance, risk mitigation strategies
Bi and Liang (2022)	China	E-commerce, IoT	Firm-level deployment, sector-specific challenges
Zhou (2023)	China	State-owned enterprises	SOE governance, cloud accounting standards
Liu (2022)	China	Municipal finance	Listed/municipal enterprise risk, local regulation
Deka et al. (2024)	India / Global	Supply chain finance	Sectoral adoption, industry integration
Cerchiello and Giudici (2016a, 2016b)	Italy / EU	Banking, systemic risk	Data privacy (GDPR), network risk, EU regulatory environment
Cong (2021)	China	E-commerce	Digital platform regulation, sector strategy
Murugan (2023)	India / Global	Banking, IoT	IT infrastructure, cross-sectoral lessons
Nivetha et al. (2024)	Global (Kaggle, Europe)	Fraud/transaction analysis	Open-access data, limited regulatory focus
Chakraborty (2019)	Global	Multiple	Regulatory trends, digitization, inclusion/exclusion
Singh et al. (2024)	Global	Multiple	Bibliometric mapping of regional and sectoral adoption

This current evidence base reveals a field dominated by context-specific solutions and regulatory path dependency. The scarcity of direct comparative studies, either across countries or sectors, underscores a critical blind spot. Few studies attempt to systematically analyze how variations in regulatory frameworks, market structures, or organizational cultures mediate the adoption and impact of big data analytics in financial risk management. This gap not only limits the transferability of current findings but also highlights an urgent research agenda for future cross-jurisdictional and cross-sectoral inquiry (Zhou et al., 2019; Cerchiello and Giudici, 2016a, 2016b; Li, 2018; Wang and Wang, 2022; Zhou, 2023; Cong, 2021; Bi and Liang, 2022; Deka et al., 2024; Singh et al., 2024; Chakraborty, 2019).

Accordingly, our synthesis should be read as context-bound to Chinese and selected European banking/fintech regimes; transfer to under-represented jurisdictions and sectors may be limited unless models are externally validated and re-tuned to different legal, market, and organizational conditions.

## 6 Limitations of the study

Despite its systematic rigor and adherence to PRISMA 2020 standards, this review is subject to several inherent limitations; some reflective of broader structural gaps in the field, others a function of methodological choices and practical constraints.

First, the review’s evidence base, while diverse in methodology and sectoral reach, remains constrained by the biases and silos present in the existing literature. The overwhelming concentration of empirical studies in Chinese and select European contexts, particularly banking and fintech, limits the transferability of findings to underrepresented regions, industries, and regulatory regimes. Cross-country, cross-sector, and comparative regulatory analyses are virtually absent from the retrieved studies, precluding robust conclusions about the global generalizability or context-dependence of big data approaches for financial risk management. To mitigate this in future updates and replications, we will (i) extend retrieval to regional/non-English sources where feasible, (ii) prioritize studies that report cross-jurisdiction external validation and clearly tag jurisdictional metadata, and (iii) encourage collaborative, multi-site designs that harmonize labels and report ΔAUC/ΔF1 across countries and sectors.

Second, the rapid evolution of both big data technologies and financial risk environments means that some included studies may already be technologically outdated, especially those employing earlier machine learning architectures or proprietary datasets no longer representative of industry best practices. While the review is temporally bounded (2016–2025) to capture the field’s recent acceleration, it remains vulnerable to the inherent lag between research, peer review, and publication.

Third, the reliance on English-language, peer-reviewed journal articles indexed in Scopus and DBLP introduces inevitable publication and language bias. High-quality research published in other languages or disseminated through alternative scholarly channels (e.g., conference proceedings, industry reports, white papers) may have been excluded, potentially narrowing the analytical lens. In recognition of possible terminology bias, we tested a minimal refresh (retaining the Big Data anchor and adding “Machine Learning” and “Systemic Risk”); it produced no additional eligible papers, though some big-data-scale studies that do not self-identify with the term may remain outside our net.

Fourth, we applied the Mixed Methods Appraisal Tool (MMAT; 2018) as a structured checklist adapted to algorithmic, non-interventional studies, reporting item-level patterns and not computing composite scores. Given the heterogeneity of tasks, outcomes, and reporting, domain-specific risk-of-bias tools (e.g., PROBAST for prediction model studies, ROBINS-I for causal non-randomized designs, AMSTAR 2 for reviews) were not uniformly applicable across our corpus. Future updates may layer in PROBAST where studies explicitly develop/validate prediction models with sufficient reporting while retaining MMAT for comparability.

**Figure 7:**
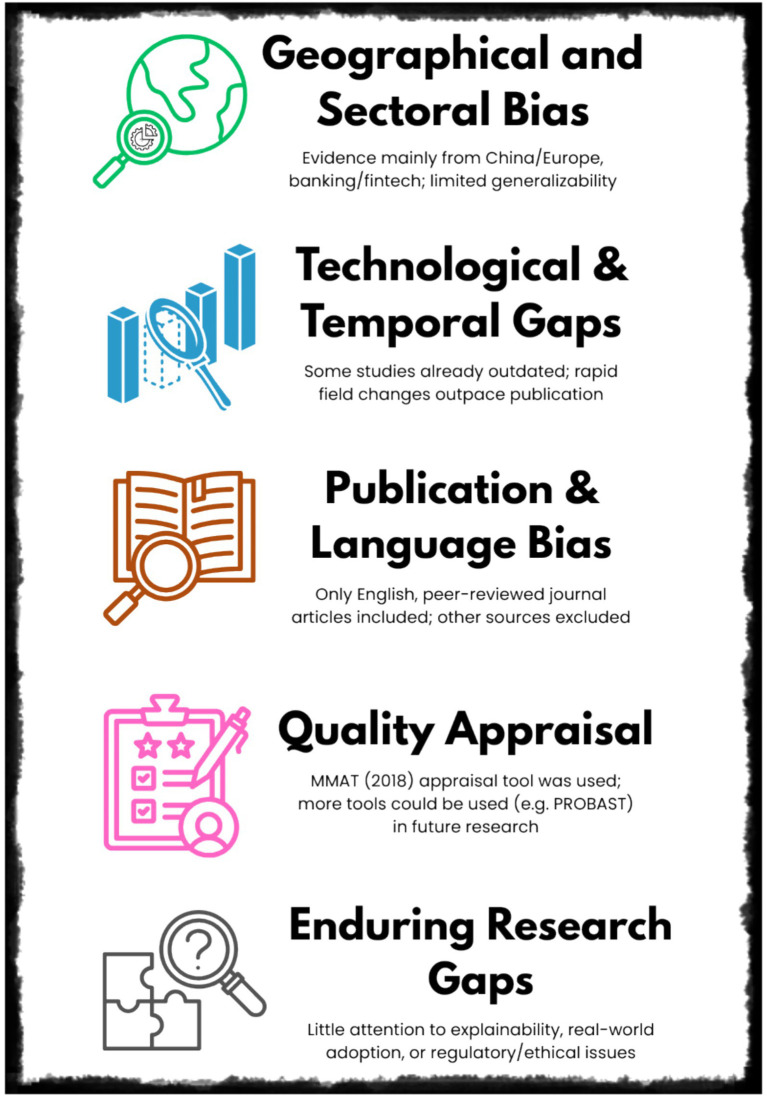
Limitations of this study.

Finally, and perhaps most fundamentally, this review is limited by the persistent blind spots in the literature itself. Despite repeated calls for attention to explainability, interpretability, managerial usability, and practical adoption, few empirical studies operationalize these dimensions in a manner that supports systematic synthesis or actionable guidance. The absence of robust, real-world deployment studies, especially outside major banking and fintech hubs, constrains the review’s ability to draw definitive conclusions about organizational integration and impact at scale. For similar reasons, emergent topics such as the integration of unstructured data, regulatory harmonization, and the interface of AI ethics and financial governance remain identified as critical gaps rather than domains of resolved knowledge (Figure 7).

## 7 Future research

The synthesis of current literature on big data and financial risk management reveals a rapidly advancing but unevenly distributed field, characterized by both methodological innovation and persistent blind spots. The limitations of the present review, and more importantly, the structural gaps in the evidence base, point to a series of urgent and actionable priorities for future research.

First, there is a pressing need for comparative, cross-jurisdictional, and cross-sectoral studies. Within this agenda, public finance, insurance, and non-bank financial institutions in emerging economies should be treated as priority contexts rather than peripheral cases, so that transferability is tested where it matters most. The current landscape is dominated by case studies in Chinese and select European banking and fintech contexts, with scant attention to emerging markets, public sector finance, or non-bank financial institutions. Future research should prioritize multi-country collaborations and comparative designs that explicitly examine how regulatory regimes, market structures, and organizational cultures mediate the adoption, effectiveness, and governance of big data analytics in risk management. Such studies will be crucial in moving the field beyond isolated exemplars toward transferable, context-sensitive best practices. Practically, we recommend pre-registered, multi-country protocols with shared open splits and jurisdiction tags, and an out-of-domain evaluation plan that reports ΔAUC, ΔF1, and Precision@k per country and sector. Subsequent updates to this review will broaden retrieval with sector-specific terms (e.g., tax compliance, procurement fraud, benefits-payment integrity; claims triage, underwriting leakage; microfinance, digital lending, Non-Bank Financial Institutions – NBFIs) and include policy/administration and insurance venues, as feasible, to reduce coverage gaps in emerging-market and public-sector settings.

Second, advancing the practical deployment and real-world validation of big data models remains a critical research frontier. While technical performance has improved, few studies address the full life cycle of model integration, from data infrastructure and workforce capability to end-user adoption and impact on decision quality. Experimental and longitudinal field studies, action research, and mixed-methods evaluations are needed to surface the organizational, managerial, and sociotechnical challenges that shape scalable and sustainable implementation. Special emphasis should be placed on underexplored domains such as insurance, public finance, and digital lending in developing economies.

Third, the field must move beyond technical metrics to systematically address explainability, interpretability, and ethical governance. The overwhelming focus on accuracy and predictive power risks sidelining issues of transparency, accountability, and compliance, especially as financial systems become more algorithmically mediated. Future research should develop and empirically test models and frameworks for explainable AI (XAI), risk communication, and human-in-the-loop decision support, while also probing the interplay between regulatory innovation (e.g., AI audits, algorithmic accountability) and technological change (Kumar et al., 2024). Concretely, we recommend regulatory-grade evaluations that pair performance with governance outcomes: time-to-conformity (documentation & logging readiness), human-oversight effectiveness (override calibration/Brier score), and post-market monitoring signals (incident rate, detection latency, drift alarms) under the EU AI Act’s high-risk regime; use standardized profiles (e.g., NIST AI RMF GOVERN/MAP/MEASURE/MANAGE) to make results comparable across deployments.

Fourth, operationalize cyber and data vulnerability as a design variable. Future work should specify, measure, and stress-test data exposure across the pipeline, including lineage integrity, contamination pathways, and drift under adversarial or failure conditions. This calls for benchmarks and reporting standards that couple predictive performance with resilience metrics, for example recovery time, detection latency, and robustness to distributional shift, so that vulnerability is managed alongside accuracy and explainability in high-stakes financial settings.

Fifth, the integration and operationalization of unstructured and alternative data sources demand deeper methodological and practical attention. Studies incorporating IoT, social media, and behavioral analytics remain rare and often proof-of-concept in nature. There is a need for scalable architectures and standardized protocols for multi-modal data fusion, as well as critical assessment of the value, reliability, and ethical risks posed by alternative data streams in high-stakes financial contexts (Adnan and Akbar, 2019).

Finally, future work should invest in open science practices and transparent reporting standards. The creation and dissemination of reproducible code, open-access datasets, and standardized evaluation benchmarks will accelerate field-wide learning and comparative analysis, helping to close current gaps in coverage, quality, and global relevance (Nurunnabi, 2021) (Table 9).

**Table 9:** Future research directions in big data and financial risk management.

Priority area	Core recommendation/research need
Comparative and Cross-Sectoral Studies	Expand to multi-country, multi-sector, and regulatory comparative designs
Real-World Deployment and Validation	Focus on implementation, adoption, and longitudinal field studies in diverse contexts
Explainability, Interpretability, and Ethical Governance	Develop and empirically test XAI, risk communication, and human-in-the-loop frameworks
Cyber and Data Vulnerability as a Design Variable	Specify, measure, and stress-test data exposure across the pipeline, including lineage integrity, contamination/poisoning pathways, and drift
Alternative and Unstructured Data Integration	Advance scalable methods and protocols for multi-modal fusion (IoT, social media, behavioral data)
Open Science and Reporting Standards	Promote open datasets, reproducible code, standardized benchmarks and clearer reporting

Building on these gaps, the following focused questions arise directly from the 21-paper corpus.

- External validity across jurisdictions. When a credit-default or bankruptcy model is trained in one jurisdiction and evaluated in another with similar features, what is the out-of-domain performance drop (e.g., ΔAUC, ΔF1), and which feature families travel? Design: pre-register splits by country or regulatory bloc; report both relative and absolute deltas. This follows the geographic concentration we observed and the lack of cross-site testing.
- Integrity and resilience of fraud models. Under controlled label noise or data poisoning at the ingestion stage, how quickly do monitoring systems detect degradation and how fast do models recover after rollback or retrain? Outcomes: detection latency, recovery time, and ΔAUC at fixed false-positive cost. This operationalizes “data vulnerability” we flagged.
- Multimodal early-warning for systemic risk. Do graph + text features (exposures, news, filings) improve early-warning precision at top-k events versus tabular baselines? Outcomes: Precision@k, lead-time in days. Many papers use single-modality tabular data; few fuse unstructured signals.
- Explainability to decision quality. Do SHAP or counterfactual explanations improve human override quality and calibration in credit or fraud review teams compared with score-only dashboards? Outcomes: Brier score, net benefit, time-to-decision in a randomized user study. This moves explainability from principle to measured effect.
- Cost-aware thresholds under class imbalance. Given realistic base rates and asymmetric loss, which thresholding schemes (cost curves, expected utility, conformal risk control) maximize net benefit across datasets commonly used in the literature? Report decision curves rather than accuracy alone; many included studies optimize symmetric metrics.
- Benchmarking fuzzy/NN/ensemble methods head-to-head. On the same open splits for the same task, which family wins where and why (e.g., IVPF-MCDM vs. deep ensembles vs. gradient boosting) when judged on accuracy and stability across time? Outcome: average rank and variance across rolling windows. Our synthesis found few true, shared-data comparisons.
- Provenance and drift. Does adding immutable data lineage and feature versioning reduce drift incidents and false positives in production risk systems over a six-month window? Outcomes: incident rate ratio and mean time between incidents; ties back to pipeline vulnerability noted in multiple studies.
- Reporting completeness and reproducibility. Is MMAT item coverage (sampling frame, outcome completeness, confounders) associated with out-of-sample performance stability across replicates? Design: correlate item-level reporting with ΔAUC across re-runs on shared splits. This leverages the appraisal patterns we documented.

Each question is tied to at least one gap we observed in the 21-paper set: limited external validation and comparability, sparse multimodal use, minimal robustness testing, and under-reporting of sampling/completeness.

## 8 Conclusion

The accelerating convergence of big data analytics and financial risk management is not merely a technological evolution; it represents a fundamental reordering of how uncertainty is apprehended and managed across financial sectors. This review, by systematically mapping and critically synthesizing 21 recent studies, exposes both the formidable advances achieved and the persistent, often underestimated limitations that continue to shape the field. It is clear that neural networks, ensemble learning, fuzzy logic, and hybrid optimization have each claimed territory in the analytics arsenal, driving measurable gains in prediction, early warning, and adaptive decision-making. Yet, these gains remain unevenly distributed, hemmed in by sectoral silos, data fragmentation, and organizational inertia.

Perhaps the most consequential insight is the field’s methodological pluralism, coupled with its persistent fragmentation. The practical trade-offs between predictive power, transparency, and deployment scalability are neither trivial nor resolved. While machine learning and AI have demonstrably raised the ceiling for what is technically possible, the persistent gap between technical validation and real-world adoption cannot be bridged by algorithms alone. The integration of non-traditional data, meaning IoT signals, behavioral analytics, sentiment streams, remains at an early, often experimental stage. Most empirical work is still bound to a narrow set of geographies, institutional logics, and regulatory frameworks, reinforcing rather than resolving questions about generalizability and impact.

This review also signals a pivotal moment for the discipline: methodological innovation alone is no longer enough. The appetite for explainability, managerial usability, and regulatory accountability is mounting, but the evidence base to support these imperatives is only just beginning to take shape. The path forward is clear; future research must confront head-on the challenges of cross-jurisdictional transferability, integration of heterogeneous data, and the operationalization of ethical, explainable analytics. Open science practices, multi-country studies, and stronger field validation are not optional luxuries, but prerequisites for the next era of financial risk management. Our closing questions in Section 7 translate the field’s generic calls into specific evaluations that can be run and compared, so progress is measurable rather than rhetorical.

In the end, big data analytics hold the promise of transforming not just the technical infrastructure of risk analysis, but the very culture of financial decision-making. To realize this promise, the field must move decisively beyond insular benchmarks and isolated pilots toward a more transparent, comparative, and practice-attuned science. Only then will big data fulfill its role as both a catalyst and a safeguard in the evolving landscape of global finance.

## Data Availability

The original contributions presented in the study are included in the article/supplementary material, further inquiries can be directed to the corresponding author.
